# Exploring causality via identification of SNPs or haplotypes responsible for a linkage signal

**DOI:** 10.1002/gepi.20236

**Published:** 2007-11

**Authors:** Joanna M Biernacka, Heather J Cordell

**Affiliations:** 1 Department of Medical Genetics, University of CambridgeUK; 2 Institute of Human Genetics, Newcastle UniversityUK

**Keywords:** fine mapping, association, conditional tests

## Abstract

In a small chromosomal region, a number of polymorphisms may be both linked to and associated with a disease. Distinguishing the potential causal sites from those indirectly associated due to linkage disequilibrium (LD) with a causal site is an important problem. This problem may be approached by determining which of the associations can explain the observed linkage signal. Recently, several methods have been proposed to aid in the identification of disease associated polymorphisms that may explain an observed linkage signal, using genotype data from affected sib pairs (ASPs) [Li et al. [2005] Am. J. Hum. Genet. 76:934–949; Sun et al. [2002] Am. J. Hum. Genet. 70:399–411]. These methods can be used to test the null hypothesis that a candidate single nucleotide polymorphism (SNP) is the sole causal variant in the region, or is in complete LD with the sole causal variant in the region. We extend variations of these methods to test for complete LD between a disease locus and haplotypes composed of two or more tightly linked candidate SNPs. We study properties of the proposed methods by simulation and apply them to type 1 diabetes data for ASPs and their parents at candidate SNP and microsatellite marker loci in the Insulin (*INS*) gene region. *Genet. Epidemiol*. 31:2727–740, 2007. © 2007 Wiley-Liss, Inc.

## INTRODUCTION

Genetic mapping studies often reveal a region of linkage containing a number of disease-associated polymorphisms. A marker may be associated with the disease either because it has direct influence on disease susceptibility (i.e. it is a “causal” polymorphism), or because it is in linkage disequilibrium (LD) with a causal polymorphism. Distinguishing polymorphisms that may be directly associated with the trait from those that are indirectly associated due to LD with a causal variant is an important problem. This problem may be addressed by trying to identify the polymorphism(s) that can explain an observed linkage result. If a particular locus is the only causal polymorphism in the region, then association with this locus should be able to explain all the linkage in the region. On the other hand, if the variant is not the causal variant, or is not the only causal variant in the region, evidence of linkage should exceed that explained by the association with this variant. Therefore, making use of genetic family data to extract both linkage and association information facilitates modelling of effects at the underlying causal loci, rather than simply detecting those effects.

A few recent studies have attempted to assess whether association with a given polymorphism is responsible for an observed linkage signal for a particular complex disease [Boutin et al., 2003; Dunn et al., 2006; Larkin et al., 2006]. However, there is no consensus on the best method to approach this problem, and generally ad-hoc methods based on subgroup analyses are applied. Furthermore, the methods proposed thus far are limited to testing whether association with a single polymorphism can account for the observed linkage. Clearly, improved methods are needed for addressing the question of whether association with a specific polymorphism or combination of polymorphisms can explain an observed linkage result.

Several methods have been proposed that may help identify polymorphisms that cause an observed linkage signal. Some of these methods focus on testing the null hypothesis that a particular variant explains none of the linkage versus the alternative hypothesis that it can explain some or all of the observed linkage in the region [Horikawa et al., 2000; Dupuis and van Eerdewegh, 2003; Li et al., 2004; Chen et al., 2005; Houwing-Duistermaat et al., 2005]. The null hypothesis can then be rejected if the candidate variant is causal or if it is in LD with a causal variant. Other methods have been proposed for testing the null hypothesis that a particular variant can explain all of the linkage in the region versus the alternative that it cannot [Sun et al., 2002; Dupuis and van Eerdewegh, 2003; Li et al., 2005]. In that case, rejection of the null hypothesis leads the investigator to the conclusion that other relevant polymorphisms exist in the region. Several methods assess linkage in subsets of data selected based on parental or children's genotypes [Horikawa et al., 2000; Dupuis and van Eerdewegh, 2003; Boutin et al., 2003]. These types of methods can exclude much of the data and may therefore be inefficient. Potentially more efficient methods model linkage conditional on parental or children's genotypes [Sun et al., 2002; Dupuis and van Eerdewegh, 2003] or by using parental or children's genotypes as a covariate in the linkage model [Houwing-Duistermaat et al., 2005].

The method proposed by Sun et al. [2002] is based on the observation that if a particular locus is the only causal variant in the region, then conditional on the genotypes at that locus for the affected individuals, there should be no unexplained identical-by-descent (IBD) oversharing in the region among the affecteds. They showed that under the null hypothesis that the candidate single nucleotide polymorphism (SNP) is the sole causal site in the region, the IBD sharing distribution of affected sib pairs (ASPs) at the candidate SNP, given their genotypes at this SNP, is independent of their affected status and depends only on their genotypes at the SNP. On the basis of this property, Sun et al. [2002] proposed test statistics similar to the usual allele-sharing-based linkage statistics, including the non-parametric linkage (NPL) statistic [Kruglyak et al., 1996] and the Zlr statistic [Kong and Cox, 1997].

In contrast to methods that evaluate linkage conditional on association, Li et al. [2005] jointly modelled linkage and association in a region. Assuming a single causal variant in the region of linkage, Li et al. [2005] proposed an approach to quantify the degree of LD between a candidate SNP and the putative disease locus. They modelled the likelihood of the marker data conditional on the trait data for a sample of ASPs, with disease penetrances and disease locus-SNP haplotype frequencies as parameters. They proposed two likelihood ratio tests to characterize the relationship of the candidate SNP and the disease locus. In contrast to typical association analysis methods that are designed to detect a relationship between an observed variant and the phenotype, but which do not reveal the pattern of LD with a possibly unobserved causal variant, the methods proposed by Li et al. [2005] for joint modelling of linkage and association are designed to model this LD pattern. The approach originally described by Li et al. [2005] does not make use of parental genotype data (even when available), and is restricted to testing whether a single SNP can explain the observed linkage. A recent implementation of this method in the software LAMP (http://www.sph.umich.edu/csg/abecasis/LAMP/), however, does utilize parental genotype data and can be used to test whether association with a microsatellite marker can explain the observed linkage.

In this paper, we describe tests of whether a haplotype composed of two tightly linked SNPs can explain all the linkage in a region. We begin by reviewing the methods of Li et al. [2005] and Sun et al. [2002] for assessing whether an observed linkage signal can be explained by the association with a single candidate SNP. Using simulations we compare the methods proposed by Li et al. [2005] and Sun et al. [2002], as well as alternatives that condition on parental candidate SNP genotypes, and demonstrate that conditioning on parental genotypes does not usually lead to large power loss. We then extend these alternative methods to test the null hypothesis that association with a haplotype can fully account for the observed linkage. We study properties of these haplotype tests by simulation. Finally, we apply a number of the methods to data for the *INS* gene associated with type 1 diabetes.

## METHODS

### JOINT MODELLING OF LINKAGE AND ASSOCIATION: “LI” AND “LI-CPG” METHODS

Recently, Li et al. [2005] proposed a method for identifying SNPs responsible for a linkage signal. Assuming there is one causal SNP in the region, they modelled the likelihood of the sibs' genotypes at markers and a candidate SNP, conditional on the sibs' affected status, in terms of the penetrances of the corresponding disease-locus genotype and disease-SNP-candidate-SNP haplotype frequencies. By restricting these haplotype frequencies appropriately, models corresponding to linkage equilibrium (LE) or complete linkage disequilibrium (LD) can be fit. Likelihood ratio statistics can then be constructed to test whether the candidate SNP and disease gene are in LE, or whether the candidate SNP and the disease gene are in complete LD, implying that either the candidate SNP or a polymorphism in complete LD with it may account fully for the linkage signal. Li et al. [2005] propose evaluating significance of these statistics by simulation. The method described by Li et al. [2005] was originally implemented in a software program called LAMA. More recently, LAMA has been replaced by the program LAMP [Li et al., 2006] (http://www.sph.umich.edu/csg/abecasis/LAMP/), which has extended capabilities including the use of parental genotype data and different types of pedigree structures. Also LAMP has improved speed and efficiency, as the *P* values are calculated using asymptotic arguments rather than by simulation. This program can perform tests of linkage, association, and tests of whether association with a particular marker can explain the observed linkage. Unlike the original method of Li et al. [2005], marker allele frequencies can be estimated by LAMP, and need not be specified before analysis.

Alternatively, we may consider modelling linkage and association jointly with additional conditioning on the parental candidate SNP genotypes, as described in Appendix A. For a sample of ASPs and their parents genotyped at *M* markers plus a candidate SNP, we model

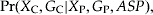

where *X*_P_ denotes the marker genotypes of the parents, *X*_C_ denotes the marker genotypes of the sibs, and GP and GC denote the candidate SNP genotypes of the parents and sibs, respectively. In Appendix A we show that this likelihood can be parameterized in terms of two relative risk parameters:

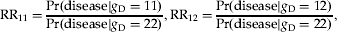

where *g*_D_ is the genotype at the disease locus, and two LD parameters:

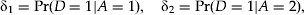

where *D* and *A* represent alleles on a disease SNPcandidate SNP haplotype. These LD parameters describe the conditional haplotype frequencies, that is, the probability of the high-risk allele ‘1’ at the disease locus, given the allele at the candidate SNP on the haplotype. If allele ‘1’ at the candidate SNP always occurs on haplotypes with allele ‘1’ at the disease SNP, then δ_1_ = 1 and δ_1_ = 0, whereas if allele ‘2’ at the candidate SNP always occurs on haplotypes with allele ‘1’ at the disease SNP then δ_1_ = 0 and δ_1_ = 1. This likelihood does not require the prespecification or estimation of marker or candidate SNP allele frequencies. We use a likelihood ratio statistic to test the null hypothesis that the candidate SNP is the sole causal polymorphism, or is in complete LD with the sole causal polymorphism in the region, and therefore association with the candidate SNP can fully account for the linkage signal. We define “complete LD” as the situation of one-to-one correspondance between the alleles at these two SNPs on a haplotype, i.e. (δ_1_, δ_2_) = (1,0) or (δ_1_, δ_2_) = (0,1). In terms of the widely used LD parameters *D*′ and *r*^2^, our definition of complete LD implies that *D*′ = 1 and *r*^2^ = 1.

We refer to this approach as Li-cpg (cpg denotes conditional on parental genotypes). Although conditioning on parental genotypes can lead to some power loss, it can also give rise to methods more robust to departures from Hardy Weinberg Equilibrium and population stratification. Furthermore it has the advantage of eliminating the requirement for allele/haplotype frequency estimation. We estimate empirical *P* values for the Li-cpg likelihood ratio statistic by simulation, as described in Appendix A.

### MODEL-FREE TESTS OF LINKAGE CONDITIONAL ON GENOTYPES AT CANDIDATE LOCI: “SUN” AND “SUN-CPG” METHODS

The method proposed by Sun et al. [2002] is based on the fact that under the null hypothesis that the candidate SNP is the sole causal site in the region,

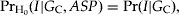
(1)
where *I* is the IBD sharing at the candidate locus and *G*_C_ are the sibs' genotypes at this locus. Sun et al. [2002] then use the distribution of ASP IBD sharing given the sibs' genotypes at the candidate SNP, *G*_C_, (which depends on allele frequencies at the SNP) to obtain

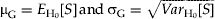

for each sib pair, for some IBD sharing statistic S. A variation of the usual NPL score statistic of Kruglyak et al. [1996] or the linear or exponential likelihood of Kong and Cox [1997] based on the standardized family score statistics

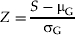

is then used to assess evidence against H_0_.

We propose modifying the method of Sun et al. [2002] by conditioning on parental (in addition to children's) genotypes, which avoids having to specify allele frequencies in the analysis. In the modified model, *G*_C_ in equation (1) is replaced by {*G*_P_,*G*_C_}, i.e. both the parental and sibs' genotypes, and therefore μ_G_ and σ_G_ are based on the IBD distribution given ASP and parental candidate SNP genotypes. We refer to this modified version of the method as Sun-cpg. Note that the Li-cpg and Sun-cpg approaches can only be used to analyze affected sib pairs with both parents genotyped at the candidate SNP, although missing marker data is allowed.

### EXTENSION OF LI-CPG TO PHASE-KNOWN HAPLOTYPES OR MULTI-ALLELIC CANDIDATE POLYMORPHISMS IN LD WITH A SINGLE CAUSAL SNP

Suppose a number of candidate SNPs are tested using the procedures described above and for each one we can reject the null hypothesis that the SNP is in complete LD with the sole causal SNP in the region, i.e. each SNP does not fully explain the observed linkage peak. Assuming there is a single causal SNP in the region, we may then ask whether association of disease with a haplotype composed of two candidate SNPs can fully explain the observed linkage, due to complete LD of the haplotype with a single untyped causal SNP. We assume the two candidate SNPs are very tightly linked and therefore there is no recombination between them, so that the two-SNP haplotype can be thought of as a single marker with four possible alleles. Since extension of the above method to a phased 2-SNP haplotype is equivalent to extending the method to a candidate locus with four alleles, we consider those two scenarios together. We describe the method only for two-SNP haplotypes or four-allele markers to simplify notation, noting that extension to markers with any number of alleles or haplotypes composed of more SNPs is straightforward. We first consider an extension of the joint model for linkage and association with conditioning on genotypes at the candidate locus (i.e. an extension to Li-cpg).

We assume there is only one causal SNP (*D*) in the region, and test whether a haplotype composed of two tightly linked candidate SNPs is in complete LD with *D*. Let RR_11_ and RR_12_ be the two relative risk parameters, as before, and let δ_i_ = Pr(*D* = 1∣*M* = *i*) for *i* = (1, …, 4), where *D* represents an allele at the disease locus and *M* represents either a two-SNP haplotype (where haplotypes 11, 12, 21, and 22 are denoted by 1, 2, 3, and 4, respectively), or a multiallelic candidate marker (with alleles 1–4). The likelihood is the same as in the case of a single candidate SNP (see equations (A.1) and (A.2)), except Pr(*D*_C_∣*G*_C_, *I*_D_, *G*_P_) is now a function of the two RR parameters and all four δ parameters. Under the null hypothesis of complete LD the δ's are all 0 or 1, such that not all are 0, and not all are 1. For example, if the disease allele (say *D* = 1) only and always occurs on haplotype 12 (*M* = 2), then δ_1_ = 0, δ_2_ = 1, δ_3_ = 0, and δ_4_ = 0. In that case, the *M* haplotype/marker fully determines the risk of disease (for that region) and fully explains the linkage signal. As before, the test is carried out by fitting the general model as well as the restricted model under the null hypothesis and calculating the likelihood ratio statistic.

Although the above discussion is fully generalizable to a marker (or haplotype) with any number of alleles, the estimation would become more difficult as the number of parameters increases. The method described above would be useful for testing whether a multi-allelic marker is in complete LD with a single causal SNP in the region; however, it would not be as useful for analyzing haplotypes, since it assumes that haplotypes are known. Discarding families for which haplotypes could not be phased could lead to a large loss of information and potential bias [Dudbridge et al., 2000]. This motivates an extension of the above method to the general situation of phase-unknown two-SNP genotypes.

### EXTENSION TO TWO-SNP GENOTYPES WITH POSSIBLY UNKNOWN PHASE: HAPLOTYPE EXTENSION TO LI-CPG

As in the description of Li-cpg, let *X*_P_ denote the marker genotypes of the parents, *X*_C_ denote the marker genotypes of the sibs, *D*_C_ denote the (unknown) disease-locus genotypes of the sibs, and ID denote the (possibly unknown) extended IBD sharing by the ASP at the candidate SNP, which equals the extended IBD sharing by the ASP at the disease-locus. Now let *G*_P_ and *G*_C_ denote the unphased genotypes of the parents and children at the two candidate SNPs. In addition, let *h*_p_ and *h*_c_ denote the phased two-locus candidate SNP genotypes of the parents and children. (Note that here “candidate” refers to a combination of two SNPs potentially in complete LD with a single unknown disease SNP, but not disease SNPs themselves. For a haplotype tightly linked to a disease SNP, “complete LD” refers to a situation such that the high risk disease SNP allele occurs only on one subset of the candidate SNP haplotypes, while the low risk allele occurs only on the remaining subset of haplotypes). Although we may be able to infer *h*_p_ and *h*_c_ for some families, these would generally be unknown and can only be determined probabilistically. Let {*H*_P_, *H*_C_} denote the set of all possible phased two-locus genotypes consistent with the parental SNP genotype data *G*_P_.

We now consider estimation of the parameters RR_11_, RR_12_, and (δ_1_, …, δ_4_), by modelling the likelihood of the data, as in the single candidate SNP case (equation (A.1)), however with *G*_P_ and *G*_C_ defined as the two-SNP genotypes. The likelihood contribution for each family is


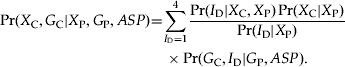


However, now,


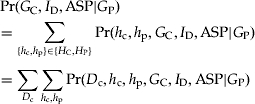


As in the single candidate SNP case (see Appendix A), we evaluate significance of the likelihood ratio test statistic by simulation. In this case we fix the genotypes of the ASPs and parents at both candidate SNPs comprising the haplotype, and sample the IBD configurations for all ASPs at the candidate SNP conditional on these genotypes. We then generate marker data for the children, given the marker IBD status and parental genotypes at the markers.

Although we have considered the question of whether association with either a single SNP, or a microsatellite, or a haplotype composed of multiple SNPs can explain all the linkage in the region, all of the models we have developed thus far assumed a single causal SNP potentially in complete LD with the candidate SNP/microsatellite/haplotype. However, existence of multiple causal variants in tight linkage is plausible, for instance if any of several mutations within a single gene, or combinations of those mutations, alter disease susceptibility. The possibility of multiple tightly linked causal polymorphisms leads to complications in our parametric modelling framework. The assumption made in the model that there is a single underlying causal SNP essentially amounts to fitting a simplified model under which there are two classes of haplotypes (low and high risk) leading to three different genotype risks. Nevertheless, because we evaluate significance using the described simulation procedure, in which all candidate loci being tested are fixed at their observed values (and therefore haplotypes composed of these SNPs are fixed), our method is expected to be valid for tests of whether association with these candidate loci can account for all the linkage in the region, regardless of how many of these SNPs are causal.

Although explicit models that allow for multiple causal SNPs that explain all the linkage in a region could be considered, difficulty arises if genotype effects and LD are modelled explicitly via the RR and δ parameters. If effects of multiple causal SNPs are allowed, the number of both the RR and the δ parameters increases substantially. In addition, explicit assumptions about the number of causal SNPs in the region must be made to fit the model. As an alternative to explicit joint modelling of linkage and association, we therefore considered an extension of the method introduced by Sun et al. [2002] for a scenario with potentially multiple tightly linked causal variants in a region.

### HAPLOTYPE EXTENSIONS OF SUN-CPG

We implemented a haplotype extension of the “Sun-cpg” approach for microsatellite markers or multiple candidate polymorphisms in a region possibly forming a haplotype (for now only implementing the NPL-type statistic). Note that this is different from the haplotype extension to Li-cpg described above, which assumed a single causal SNP in the region. Here we are not making such an assumption. The method proceeds exactly as the Sun-cpg for the single-SNP case (i.e. in equation (1) *G*_C_ is replaced by *G*_C_, *G*_P_), except now we calculate Pr(I∣*G*_C_, *G*_P_), where *G*_C_, *G*_P_ are the genotypes of parents and children at *two or more* tightly linked candidate SNPs. To calculate these quantities, we use a new version of Merlin [Abecasis and Wigginton, 2005] that can estimate IBD sharing given a number of markers, taking into account LD within haplotype blocks.

## RESULTS

### SIMULATIONS

We used the models listed in Table I to study type 1 error and power of methods for testing whether associations with a candidate SNP can fully account for an observed linkage signal. For Models 1–4, the second SNP in the haplotype was the sole causal polymorphism in the region. We carried out tests of whether association with the first SNP in the haplotype could explain the observed linkage. This first SNP in the haplotype (the “candidate SNP”) is either in complete LD with the causal SNP (therefore association with this candidate SNP can fully explain the linkage—the null hypothesis is true) or is in incomplete LD (the null hypothesis is not true). Different levels of LD are considered within each disease-generating model to demonstrate the effect of decreasing LD between the disease and candidate SNP on power. For Model 5, three SNPs in a haplotype influence disease susceptibility; thus the null hypothesis is not true. All models follow a multiplicative disease risk model, except for Model 4. ASP and parental genotypes were generated at five markers spaced at 2.5cM intervals (i.e. at 0.0, 2.5, 5.0, 7.5, and 10.0 cM). Each marker had four equally frequent alleles. The disease and candidate SNPs were located at 5.2cM along this map, these two loci being fully linked but with varying levels of LD. Parental affected status was treated as unknown.

**TABLE I:** Simulation models:single SNP analysis

			LD			
					
Model	Description	Risk contributions for haplotypes(11,12,21,22)^a^	*D′*	*r^2^*	Frequency of haplotypes(11,12,21,22)	Disease prevalence
Model 1—full LD	Multiplicative	(0.15,0.30,0.15,0.30)	1.00	1.00	(0.70, 0.00, 0.00, 0.30)	0.038
Model 1—high LD			0.74	0.44	(0.60,0.05,0.10,0.25)	
Model 1— mid LD			0.44	0.13	(0.50,0.10,0.20,0.20)	
Model 1—low LD			0.23	0.025	(0.40,0.12,0.30,0.18)	
Model 2—full LD	Multiplicative	(0.10,0.30,0.10,0.30)	1.00	1.00	(0.50,0.00,0.00,0.50)	0.040
Model 2—high LD			0.80	0.64	(0.45,0.05,0.05,0.45)	
Model 2—mid LD			0.52	0.27	(0.38,0.12,0.12,0.38)	
Model 2—low LD			0.20	0.04	(0.30,0.20,0.20,0.30)	
Model 3—full LD	Rare disease allele	(0.10,0.30,0.10,0.30)	1.00	1.00	(0.95,0.00,0.00,0.05)	0.0121
Model 3—full LD			0.70	0.05	(0.65, 0.01, 0.30,0.04)	
Model 4—full LD	Non-multiplicative	(0.01, 0.01,0.05)	1.00	1.00	(0.70,0.00,0.00,0.30)	0.0136
Model 4—high LD			0.67	0.44	(0.63,0.07,0.07,0.23)	
Model 4—mid LD			0.52	0.27	(0.60,0.10,0.10,0.20)	
Model 5	Causal haplotype	(0.10,0.15,0.15,0.20,0.15,0.20,0.20,0.30)			(0.20,0.15,0.05,0.10,0.10.,0.05,0.15,0.20)	0.0342

a Risks are calculated by multiplying td risk contributions of a person's two haplotypes, except for Model 4. For Model 4, td table shows the genotype risks for genotypes 11, 12, 22 at the second SNP in the haplotype. Under Model 5, there are three tightly linked diseasesusceptibility SNPs forming a haplotype. For this model, the table shows the haplotype risk and frequencies for haplotypes 111, 112, 121, 122, 211, 212, 221, 222.

Results in Table II demonstrate td under td null hypothesis (full LD), correct type 1 error rates were achieved with all methods except the LAMP test of complete LD which gave conservative results in the situations investigated in our simulations. As expected, power increased as the sample size increased and as the level of LD between the candidate SNP and the causal SNP decreased. Under most of the multiplicative models considered (see Models 1, 2, and 5), the method proposed by Sun et al. [2002] was most powerful. A similar approach with additional conditioning on parental candidate marker genotypes, which does not require allele frequency specification, led to some power loss. However, the method of Sun et al. [2002] can be highly sensitive to misspecification of these parameters. For Model 2, the true candidate SNP high-risk allele frequency is 0.5. In the simulations presented in Table II, correct allele frequencies were used. When analysis was performed assuming an allele frequency of 0.45, the type 1 error of the Sun et al. [2002] method rose to 26.3%. When the data was re-analyzed assuming an allele frequency of 0.55, the type 1 error dropped to about 0%, with the power dropping to 6.9, 44.6, and 83.1% for the high, medium and low LD models. Therefore, although the Sun-cpg method generally has lower power than the approach proposed by Sun et al. [2002], when allele frequency estimates may be inaccurate, this approach has a considerable advantage. Conditioning on parental genotypes at the candidate SNP, in addition to those of the sibs, negates the need for specifying allele frequencies and therefore leads to a more robust test.

**TABLE II:** Simulation results: single SNP analysis

		Type 1 error/power^a^			
		
Model	Sample size	LAMP-LD^b^	Li-cpg	Sun	Sun-cpg
Model 1—full LD	500	0.015	0.044	0.054	.047
Model 1—high LD	500	0.118	0.145	0.199	0.191
Model 1—mid LD	500	0.253	0.220	0.367	0.327
Model 1—low LD	500	0.324	0.275	0.447	0.371
Model 1—full LD	1,000	0.006	0.058	0.052	0.064
Model 1—high LD	1,000	0.194	0.280	0.327	0.288
Model 1—mid LD	1,000	0.483	0.495	0.602	0.531
Model 1—low LD	1,000	0.608	0.530	0.704	0.607
Model 2—full LD	500	0.019	0.058	0.040	0.041
Model 2—high LD	500	0.296	0.260	0.277	0.240
Model 2—mid LD	500	0.699	0.680	0.732	0.646
Model 2—low LD	500	0.848	0.775	0.903	0.833
Model 2—full LD	1,000	0.022	0.044	0.046	0.051
Model 2—high LD	1,000	0.545	0.435	0.428	0.404
Model 2—mid LD	1,000	0.961	0.895	0.929	0.879
Model 2—low LD	1,000	0.993	0.990	0.990	0.976
Model 3—full LD	1,000	0.016	.040	0.049	0.050
Model 3—mid LD	1,000	0.797	0.708	0.757	0.641
Model 4—full LD	1,000	0.023	0.043	0.039	0.037
Model 4—high LD	1,000	0.999	0.976	0.917	0.868
Model 4—mid LD	1,000	0.999	0.994	0.992	0.981
Model 5	1,000	0.232	0.251	0.366	0.308

a For Li-cpg, type 1 error estimates are based on 500 data replicates, and power estimates are based on 200 data replicates. For all other methods type 1 error and power estimates are based on 1,000 replicates. When data are generated under “full” LD, the null hypothesis is true, and values in the table are estimates of type 1 error for a test of nominal size 0.05.

b LAMP-LD is the test for complete LD implemented in the software LAMP.

Under the simulated model with a rare high-risk allele (Model 3) and the non-multiplicative model (Model 4), the LAMP test for complete LD was more powerful than the Sun approach. Again, conditioning on parental genotypes generally led to some power loss, although in most cases the power loss was not high. In fact, in some of the low–power scenarios (e.g. Model 1—high LD), Li-cpg was more powerful than the test of complete LD implemented in LAMP. The program LAMP requires specification of the prevalence, and estimates allele frequencies from the data. In our simulations we observed that misspecification of the prevelance can reduce power (data not shown). However, prevalence estimates are usually quite reliable, so this is not a major concern. When there is more than one causal SNP in a small region, the assumption of a single causal SNP in the region made by the LAMP and Li-cpg methods is violated. Under a model with a causal haplotype made up of three SNPs, all of which influence disease susceptibility (Model 5), we found that the Sun and Sun-cpg approach were more powerful than Lamp and Li-cpg, presumably because they do not make the incorrect assumption of a single causal SNP.

We also studied the performance of our haplotype extensions of the methods by simulation under the models shown in Table III. For models “Null 1”, “Alt 1”, and “Alt 2” the haplotype risk only depends on the allele at the third locus, and therefore the third locus is the sole causal SNP. Under model “Null 1” the loci 1–2 haplotype is in complete LD with the third locus, and therefore the null hypothesis is true. Under models “Alt 1” and “Alt 2” the candidate haplotype is not in complete LD with the causal SNP, and therefore the alternative hypothesis is true. Under the “Null 2” model, association with loci 1 and 2 explains all the linkage, but not because the loci 1–2 haplotype is in complete LD with the sole causal SNP in the region, but rather because loci 1 and 2 are the only two causal SNPs in the region. This model is used to test the sensitivity of the extension of Li-cpg to the assumption of a single causal SNP in the region.

**TABLE III:** Simulation models: haplotype analysis

Model	Haplotype risks^a^	LD *D*′^b^	Haplotype frequencies
Null 1	(0.1,0.3,0.1,0.3,0.1,0.3,0.1,0.3)	1.00	(0.20.0.00,0.15,0.00,0.15,0.00,0.50)
Null 2	(0.1,0.1,0.2,0.2,0.2,0.2,0.4,0.4)	1.00	(0.00,0.25,0.00,0.25,0.00,0.25,0.00,0.25)
Alt 1	(0.1,0.3,0.1,0.3,0.1,0.3,0.1,0.3)	0.39	(0.10,0.05,0.10,0.05,0.10,0.05,0.15,0.40)
Alt 2	(0.1,0.3,0.1,0.3,0.1,0.3,0.1,0.3)	0.17	(0.09,0.06,0.09,0.06,0.09,0.06,0.25,0.30)

a aRisks are calculated by multiplying the risk contributions of a person's two haplotypes. Risks and frequencies are given for haplotypes (111, 112, 121, 122, 211, 212, 221, 222).

b bHere D0 represents Hedrick's D′ measure of LD for multi-allelic markers [Hedrick, 1987]. We use it to represent the LD between the loci 1 and 2 haplotype (treated as a four-allele marker) and the third locus, which is the disease SNP. Under the “Null 2” model the loci 1–2 haplotype is itself causal (rather than locus 3). Therefore in this case we report D0 between the candidate loci 1–2 haplotype and the causal (loci 1–2) haplotype, which is clearly *D*′=1.

Results are shown in Table IV. Table IV also shows the average Kong and Cox [1997] LOD score obtained when testing for initial linkage. In simulations these methods gave type 1 errors close to the nominal 5%. Note that under the “Null 2” model both loci in the haplotype tested are causal, so that the assumption of a single causal SNP in LD with the candidate haplotype made by the Li-cpg haplotype method is violated. However, the type 1 error is still correct, demonstrating that the method is robust to failure of this assumption. This is because of the way significance of the statistic is assessed by a simulation procedure which fixes all the candidate SNP genotypes, as discussed in Appendix A.

**TABLE IV:** Simulation result:haplotype analyses

				Type 1 error/power^a^	
				
				Haplotype extension of	
				
Model	Sample size	LD level	Kong and Cox LOD score	Li-cpg	Sun-cpg^b^
Null 1	500	Full	2.75	0.056	0.036
Null 2	500	Full	2.46	0.056	0.034
Alt 1	500	Mid	3.04	0.44	0.43
Alt 1	1,000	Mid	5.70	0.73	0.80
Alt 2	500	Low	2.36	0.62	0.70
Alt 2	1,000	Low	4.41	0.95	0.92

a Type 1 error estimates for a test of nominal size 0.05 are based on 500 data replicates. Power estimates are based on 100 data replicates. When td null td is true, the values in the table are estimates of type 1 error.

b td extension of Sun-cpg td for haplotypes used here is based on the NPL-type statistic with weights=σ*_G_*.

Here the same marker map was used as for simulations in Table II (markers with four equally frequent alleles at 0.0, 2.5, 5.0, 7.5, 10.0cM; candidate and disease SNPs at 5.2 cM).

On td basis of td simulation results presented in Table IV, it appears that extensions of the Li-cpg and Sun-cpg approaches to haplotypes have similar power. We expect the haplotype tests to be less powerful than the corresponding single-SNP tests because of the presence of additional LD parameters (although power cannot be compared directly, as the tests address different hypotheses). This reduction in power may possibly lead to a requirement for substantial evidence of linkage in the region, for the test to be useful in practice. Nevertheless we find that even with the simulated models with moderate levels of linkage, the tests have reasonably good power. Under the “Null 1” Model, with a sample of 1,000 ASPs, the single-SNP Sun-cpg approach has 54% power to reject each of the two candidates as the sole causal locus, while the Sun-cpg haplotype approach has the correct 5% type 1 error for the hypothesis that the haplotype is in complete LD with the sole causal locus. Under the “Alt 1” Model, with a sample of 1,000 ASPs, the single-locus Sun-cpg method has approximately 95% power to reject each of the two candidate SNPs as the sole causal locus, while the Sun-cpg haplotype method has 80% power to conclude that the haplotype composed of the two SNPs is not in full LD with the sole causal locus.

### APPLICATION TO TYPE 1 DIABETES DATA

The methods described in this paper were applied to study the effects of polymorphisms in the insulin gene (*INS*) region associated with type 1 diabetes [Barratt et al., 2004]. By analyzing *75* polymorphisms in the *INS* region, Barratt et al. [2004] found two equally likely candidates for the causal locus, in addition to a previously identified VNTR. Using a stepwise conditional logistic regression approach, they showed that none of the other genotyped polymorphisms contributed significantly to the risk of type 1 diabetes after accounting for either the −23*HphI* polymorphism or +1140A/C SNP. Their analysis revealed that LD with −23*HphI* is sufficient to explain the association of all the other markers tested. Further analysis showed that +1140A/C could perhaps be just as effective in explaining the observed association in this region. They concluded that susceptibility in this region could be attributable to a single polymorphism in the *INS* region. They also noted that because of the strong LD between the VNTR, −23*HphI*, and +1140A/C, resolution of these effects may not be achievable by association studies of European populations.

The analysis carried out by Barratt et al. [2004] provided no evidence for significant association at the remaining genotyped polymorphisms, after accounting for the effect of −23*HphI* or +1140A/C. However, the possibility of further unknown variants in this region contributing to type 1 diabetes had not been tested. If other untyped variants are directly associated with type 1 diabetes, they should contribute to the linkage in the region. In that case, association with either −23*HphI* or +1140A/C may not explain all observed linkage at these loci. To test whether this is the case, we applied the methods described in this paper.

Analysis of 437 ASP families genotyped for −23*HphI*, using the LAMP program (http://www. sph.umich.edu/csg/abecasis/LAMP/) suggests that association with −23*HphI* cannot explain all the linkage in the region (*P* = 0.01). Using the same approach, analysis of 317 ASP families genotyped for +1140A/C suggests association with this SNP also cannot account for all the observed linkage (*P* = 0.003). Analysis of the same two candidate SNPs with the approach of Sun et al. [2002] also leads to the rejection of the null hypotheses that one of these may be the sole causal variant in the region (*P* = 0.01 and 0.005 for −23*HphI* and +1140A/C, respectively). We note that the Sun et al. [2002] analysis required pre-specification of candidate SNP allele frequencies, and, as our simulations demonstrated, results tend to be very sensitive to misspecification of these parameters.

Having rejected the null hypothesis of direct association with LAMP, we examined the parameter estimates from LAMP analysis obtained under the indirect association model. For analysis of the −23*HphI* polymorphism, the relative risk estimates for the disease locus were 2,322 and 11,911, with an estimated attributable fraction for this locus of 0.9999. These point estimates seem unrealistic given current beliefs about genetics of complex traits, and given the fact that type 1 diabetes susceptibility can be largely attributed to genes in the HLA region. Analysis of the +1140A/C SNP using an indirect association model leads to disease locus relative risk estimates of 1.11 and 18.37, with the attributable fraction estimated at 0.905, which again seems unrealistic. Investigation of the properties of parameter estimates from LAMP, and estimation of confidence intervals for these parameters, would be of interest. These investigations are beyond the scope of this paper.

We re-analyzed the *INS* gene data using the other approaches studied in this paper. Analysis of 437 ASP families genotyped for −23*HphI*, using the approach for joint modelling of linkage and association with conditioning on parental genotypes Li-cpg), provided no evidence of linkage unexplained by association with this variant (*P* = 0.31). Using the same approach, analysis of 317 ASP families genotyped for +1140A/C provided no evidence of linkage unaccounted for by association with +1140A/C (*P* = 0.27). The model-free approach to modelling linkage conditional on candidate SNP genotypes with conditioning on parental genotypes Sun-cpg) also suggested that there is no evidence of other associations further contributing to linkage in the *INS* region after accounting for association with either −23*HphI* or +1140A/C (*P* = 0.300, and 0.296 for the two SNPs, respectively).

The difference between the results from the conditional and unconditional analyses suggest that for these particular data, conditioning on parental genotypes may be leading to a substantial power loss. However, it is also possible that the Sun and LAMP approaches are leading to false positive results due to violations of some assumptions made by these methods. For instance, methods that condition on parental genotypes are expected to be less sensitive to population stratification and departures from Hardy Weinberg Equilibrium. In the presented simulations, all model assumptions were satisfied. On the other hand, in an additional simulation with preferential sampling of heterozygous parents (results not shown), the Sun-cpg method retained correct type 1 error while the Sun approach resulted in highly inflated type 1 error.

Finally, we applied the haplotype methods described in this paper to analyze the haplotype composed of +1140A/C and −23*HphI*. Since these approaches also condition on parental genotypes, we expect they may also have insufficient power for this data set. Using our haplotype extension of the approach for joint modelling of linkage and association, analysis of 304 ASP families revealed no evidence of linkage unaccounted for by the association of type 1 diabetes with the +1140A/C −23*HphI* haplotype (*P* = 0.28). Under the null hypothesis (which could not be rejected) estimates of the d parameters for haplotypes 11, 12, 21, and 22 were 0, 1, 0, and 1, respectively, with RR_11_ estimated at 0.35, and RR_12_ at 0.55. This indicates that haplotypes 21 and 11 are the high risk haplotypes, and therefore that the ‘1’ allele at −23*HphI* is associated with increased risk of type 1 diabetes. Using our haplotype extension of the Sun-cpg approach, we also cannot reject the null hypothesis that association with the +1140A/C −23*HphI* haplotype can fully account for the observed linkage (*P* = 0.62).

### DISCUSSION

In this paper, we describe methods for assessing whether association between a candidate SNP, multiallelic marker, or haplotype composed of a number of SNPs can explain an observed linkage result. For many diseases, data consisting of ASP and parental genotypes have been collected for linkage studies, with subsequent genotyping of SNPs for fine-mapping in regions of interest. The methods described in this paper can be applied to such data. We considered the approaches proposed by Li et al. [2005] and Sun et al. [2002] and extensions of these methods. Our simulations showed that all the methods studied provided correct type 1 errors, except for the LAMP test for complete LD, which was conservative for the models we studied. Although in our simulations under multiplicative models the method proposed by Sun et al. [2002] tended to be most powerful, it can lead to highly inflated type 1 errors when allele frequencies are misspecified. LAMP [Li et al., 2005, 2006] was most powerful in our simulations under a recessive model. In our simulations we assumed that parental phenotypes were unknown. In additional simulations (results not shown) we used LAMP to analyze data with parental phenotypes, which produced a slight power increase. We also note that if simulation-based *P* value calculation was implemented in the LAMP program, the LAMP test for complete LD should no longer be conservative, and higher power should therefore be achieved. Currentlly LAMP does not have an option for calculating empirical *P* values, presumably due to the high computational demands this would introduce.

We extended the Li-cpg and Sun-cpg methods to the case of haplotypes composed of two candidate SNPs. Methods for assessing whether a haplotype can explain all the linkage in a region are important for two reasons. Even if the assumption of a single causal SNP in the region is correct, the causal variant may not have been genotyped, and a haplotype composed of two or more SNPs may be in complete LD with it even when none of the genotyped SNPs are (i.e. haplotypes may be more useful for studying indirect association). Also, in many situations, the idea of a single causal SNP in a region is unrealistic.

The model-based approaches proposed by Li et al. [2005] and extended in this paper require assumptions to be made about the number of disease polymorphisms in the region and the number of alleles at these loci. Having made the assumption of a single causal SNP, the methods provide estimates of genotype relative risk and LD parameters, which can contribute to our understanding of the possible role of the candidate SNP. Although parameter estimation is possible, simulations (data not shown) and results from the real data analysis suggest that the point estimates are generally not very accurate. The approach of Sun et al. [2002] and our extension of this method do not provide estimates of parameters that describe the underlying genetic model, with the benefit that no assumptions about the mode of inheritance are necessary. Under a model with three tightly linked causal SNPs, we found the method of Sun et al. [2002] and the related approach conditioning on parental genotypes to be more powerful than the LAMP test of complete LD and Li-cpg. The benefits of the lack of assumptions about the underlying genetic model with the Sun and Suncpg approaches extend to the haplotype analysis. Our haplotype extension of the Li-cpg approach still assumes a single underlying causal SNP that may be in complete LD with the candidate haplotype, whereas the haplotype extension of the Sun-cpg approach makes no assumptions about the underlying disease model.

As discussed by Sun et al. [2002], one of the weaknesses of their method is that sib pairs with genotypes at the candidate SNP that are highly informative in terms of IBD sharing are less informative for this method. This is because the power of the method depends largely on *E*_H_A__[*S*∣*G*_C_] − *E*_H_0__[*S*∣*G*_C_], and when *G*_C_ provides complete information on *S*, *E*_H_A__[*S*∣*G*_C_] = *S* = *E*_H_0__[*S*∣*G*_C_]. As indicated by Sun et al. [2002], when *G*_C_ provides close to complete information on *S*, power will be low. Consequently, this approach may not be very powerful when applied to microsatellite candidates or multiple tightly linked candidates. As Sun et al. [2002] indicated, this loss of power is the price paid for not making assumptions about the underlying genetic model.

All of the methods discussed in this paper can produce inflated type 1 errors if they are applied to locations chosen based on the fact that the evidence for linkage exceeds a given threshold, and the same data are subsequently used for testing whether the linkage is explained by the association. Li et al. [2005] demonstrated that their ability to detect complete LD was dramatically enhanced as the evidence for linkage increased. However, they did not point out that if those methods were applied only at locations with lod scores exceeding some threshold, elevated type 1 errors would result. This issue was addressed by Sun et al. [2002].

Analysis of the diabetes data in the *INS* gene region demonstrates the need for further investigation of these methods. Some markers used for estimation of linkage in the region in our analyses were spaced quite densely, and therefore the LE assumption may have been violated. Other potential violations of assumptions made by the different methods may have had an effect. For example, as previously discussed, misspecification of allele frequencies can lead to inflated type 1 errors for the Sun et al. [2002] method. Results of the *INS* gene data analysis indicate that examination of the properties of parameter estimates is also needed. An improved understanding of the estimator properties may help with interpretation of results from the different methods. The *INS* data example further indicates that in certain situations, conditioning on parental genotypes may lead to larger power loss than we observed in our simulations. This suggests that a haplotype extension of the approaches of Li et al. [2005] and Sun et al. [2002] without conditioning on parental genotypes may be useful. However, the approaches that do not condition on parental genotypes must be applied with greater caution, as they are expected to be less robust to failures of assumptions (e.g. Hardy Weinberg Disequilibrium). Also, the sensitivity of the method of Sun et al. (2002) to allele-misspecification would be of greater concern if the method was extended to haplotypes, as accurate haplotype frequency estimation is more difficult than accurate allele frequency estimation.

Another noteworthy approach that uses both association and linkage information for relative risk estimation is the MASC method introduced by Clerget-Darpoux et al. [1988]. This approach can also use additional information such as differential risk of being affected for specific relatives of probands, and allows the testing of goodness-of-fit of various models. However, model specification and selection with this approach is cumbersome and requires the estimation of numerous parameters. A comparison of the approaches described in this paper with the MASC method would be interesting.

Although statistical analysis of linkage and association data cannot alone establish causality, the analytical methods described in this paper can aid in distinguishing variants that may be the sole causal variants in a region, from those that are unlikely to be. The hypothesis formulation in this problem may appear rather “unusual”, because the test will never allow us to conclude that we have identified all the causal variants in the region. Therefore, when the null hypothesis is not rejected all we can say is that we have insufficient evidence to conclude that the candidate SNP is the sole causal SNP in the region. This is not surprising, since statistically, we can never accept a null hypothesis. However, it is slightly unsatisfactory, since we would prefer to be able to conclude (at a certain level of significance) that the variant(s) tested do account for all the linkage in a region—in other words to reject the null hypothesis that there are other variants in the region. Of course expecting a statistical method to have the ability to lead to a conclusion that all causal variants in a region have been identified is not reasonable. Even if the key genetic factors have been identified, there may always exist genetic variants with such minute effects on the linkage signal that they are essentially undetectable. A related approach, beyond the scope of this paper, could be used to estimate “how much” of the observed linkage is accounted for by the association. That raises the question of how to quantify the observed/accounted for linkage. Further research into such methods is warranted. Nevertheless, the methods described in this paper can be used to aid researchers in prioritizing SNPs for further study, and to inform them when genotyping of additional SNPs should be undertaken.

## APPENDIX A

### DETAILS OF THE LI-CPG METHOD

We assume an ASP design, with ASPs and their parents genotyped at *M* markers plus a candidate SNP. Similar to Li et al. [2005], we assume that the *M* markers are in LE with one another and with the candidate SNP, and there is one causal SNP in the region, closely linked to the candidate SNP, with no recombination between the two loci.We would like to test whether the candidate SNP may be the sole causal polymorphism in the region. We consider a model similar to that of Li et al. [2005], however we additionally condition on parental genotypes. This means that our likelihood does not depend on the disease locus allele frequency parameter. The likelihood contribution for each family, conditional on the parental genotypes, is

where *X*_P_ denotes the marker genotypes of the parents, *X*_C_ denotes the marker genotypes of the sibs, and *G*_P_ and *G*_C_ denote the candidate SNP genotypes of the parents and sibs, respectively. Below, we show this likelihood can be parameterized in terms of two relative risk parameters:

where *g*D is the genotype at the disease locus, and two LD parameters:

where *D* and *A* represent alleles on a disease SNP-candidate SNP haplotype. Note that these are the same LD parameters as those used by Cantor et al. [2005], however their method is designed for testing different hypotheses, and they model recombination and LD parameters, rather than association and LD parameters. Also, let *D*_C_ denote the (unknown) disease-locus genotypes of the sibs and *I*_D_ denote the (possibly unknown) extended IBD sharing by the ASP at the candidate SNP, which equals the extended IBD sharing by the ASP at the disease-locus, under the assumption of no recombination between these two loci. By extended IBD sharing we mean one of four IBD states: sharing zero alleles IBD, one allele IBD from the mother, one allele IBD from the father, or two alleles IBD. We code these four IBD states as *I*_D_ = 1, 2, 3, 4, respectively.

The likelihood contribution for each family, conditional on the parental genotypes, is

(A.1)

Pr(*I*_D_∣*X*_C_, *X*_P_) can be obtained using software such as Merlin [Abecasis et al., 1996]. Pr(*X*_C_∣*X*_P_) does not depend on the disease model parameters and cancels out when the likelihood ratio statistic is calculated.

pr (*G_C_*,*I_D_*∣*G_P_*, ASP) can be calculated as

(A.2)

Note that we have assumed that Pr(ASP∣*D*_C_, *G*_C_, *I*_D_, *G*_p_) = Pr(ASP∣*D*_C_), so that the postulated disease locus is the only causal locus in the region. We further assume that Pr(ASP∣*D*_C_) = Pr(disease∣*D*_C1_) Pr(disease∣*D*_C2_), where *D*_C1_ and *D*_C2_ are the disease locus genotypes of sibs 1 and 2, respectively. Thus we are assuming no other shared genetic or environmental causes of disease. However, Li et al. [2005] pointed out that this is a reasonable assumption when there are multiple disease-causing variants or shared environmental risk factors. Our simulations (results not shown) also show that presence of other unlinked causal genes does not appear to effect type 1 error or power. Because each term in the numerator and the denominator of the likelihood contains the terms Pr(disease∣*D*_C_1) and Pr(disease∣*D*_C_2), we may divide both the numerator and denominator by Pr(disease∣*D*_C_1 = 22) Pr(disease∣*D*_C_2 = 22). Therefore, we estimate the two relative risk parameters rather than the three penetrances. Pr(*G*_C_∣*I*_D_, *G*_p_) are constants, not depending on the disease model, which we have tabulated for a biallelic locus. Pr(*D*_C_∣*G*_C_, *I*_D_, *G*_p_) are functions of the LD parameters, δ_1_ and δ_2_, as shown below.

We may perform tests similar to those proposed by Li et al. [2005]. The likelihood for all the ASP families is the product of the individual family likelihoods. To fit this likelihood (under the alternative hypothesis) we constrain the RR parameters to be greater than or equal to 1 and the δ parameters are restricted to lie in [0,1]. To fit the likelihood under the null hypothesis, we restrict the parameters δ1 and δ2 to their null values of 0 and 1 or 1 and 0. Under the null hypothesis the candidate SNP is the sole causal polymorphism, or is in complete LD with the sole causal polymorphism in the region, and therefore association with the candidate SNP can fully account for the linkage signal. Li et al. [2005] refer to this situation of complete LD as “plausible causality”. In that case either the observed candidate locus is the causal variant, or there is a one-to-one correspondence between the alleles at the candidate and disease SNPs, such that only two haplotypes occur in the population. Rejection of complete LD for a candidate SNP suggests that this SNP cannot fully account for the observed linkage signal; there is at least one other polymorphism in the region which directly affects the trait. Because of the complexity of the parameter space, we do not derive the null distribution analytically, and suggest assessing significance empirically by simulating data under the null hypothesis. We use the following procedure to generate data under the null hypothesis.

- For each ASP, fix candidate SNP genotypes of sibs and parents at observed values. Also fix parental marker genotypes at observed values.
- Sample the IBD configuration at the candidate SNP, given the observed SNP genotypes of the ASP and their parents. (Similar to the scheme used by Li et al. [2005], but parental SNP genotypes also stay fixed.)
- Generate IBD status at markers, conditional on the IBD status at the SNP.
- Generate marker data for children, given the marker IBD status and parental genotypes at the markers.

Using the above scheme, we generate a large number of data sets, calculate the test statistic for each one, and use the resulting distribution to empirically estimate the *P* value for the test statistic obtained from the original data.

## APPENDIX B

### COMPUTATIONAL DETAILS FOR SECTION ON “EXTENSION TO TWO-SNP GENOTYPES WITH POSSIBLY UNKNOWN PHASE”

In the case of testing for complete LD between a haplotype composed of two SNPs and a single causal SNP, the numerator of

can be calculated as

We estimate Pr(*h*_c_; *h*_p_∣*G*_P_) using ZAPLO software [O'Connell, 2000], which provides all possible haplotypes, including information on the parental origin for the children's haplotypes, and their probabilities. Since we use ZAPLO to estimate the haplotype probabilities from an ascertained sample, these probability estimates may not be unbiased estimates of the true probabilities. However, since we use this same procedure to analyze all the simulated data generated under the null hypothesis for computing the *P* values, this issue does not appear to have a negative impact on type 1 errors of our approach.

We calculate a likelihood ratio statistic and assess significance by simulation. To generate data under the null hypothesis, for each ASP we first obtain the distribution Pr(*I*_D_∣*G*_C_, *G*_P_) using a new version of Merlin that estimates IBD sharing taking into account the LD between markers [Abecasis and Wigginton, 2005]. We generate *I*_D_ from this distribution for each ASP, then generate IBD sharing at all markers, conditional on IBD sharing at the candidate haplotype, and finally marker genotypes for the sibs, conditional on the marker IBD and parental marker genotypes.
